# Supplementary material for: A comparative study of structural variant calling in WGS from Alzheimer’s disease families
Source: Life Sci Alliance. 2024 Feb 28;7(5):e202302181. doi: 10.26508/lsa.202302181 (PMC10902710; doi:10.26508/lsa.202302181)
Supplement: Supplementary file 1 [file LSA-2023-02181_TableS1.docx]

**Supplementary Tables**

**Supplementary Table 1**

| **Caller** | **File Size (GB) - Disc** | **File Size (GB) - Disc+ext** | **Avg. Run Time (hours) - Disc** | **Avg. Run Time (hours) - Disc+ext** | **Peak CPU % - Disc** | **Peak CPU % - Disc+ext** | **Peak Memory (GB) - Disc** | **Peak Memory (GB) - Disc+ext** |
| --- | --- | --- | --- | --- | --- | --- | --- | --- |
| Breakdancer | **209.05** [130.14-318.90] | **54.58** [75.32-37.29] | **4.94** [3.11-6.87] | **1.44** [0.77- 2.14] | **22.70** [14.90-30.49] | **5.67** [3.73-7.62] | **23.26** [15.15-37.11] | **6.16** [5.23-9.00] |
| CNVnator | **209.05** [130.14-318.90] | **54.58** [75.3237.29] | **1.79** [1.04-2.87] | **0.50** [0.40-0.69] | **4.43** [2.71-8.99] | **1.10** [0.78-1.60] | **2.23** [1.26-3.74] | **0.57** [0.49-0.82] |
| DELLY | **209.05** [130.14-318.90] | **54.58** [75.32-37.29] | **10.10** [6.60-16.64] | **2.78** [1.60-5.60] | **61.63** [37.58-80.95] | **16.61** [9.47-22.59] | **3.53** [2.05-5.71] | **0.96** [0.64 -1.65] |
| GATK | **209.05** [130.14-318.90] | **54.58** [75.32-37.29] | **5.86** [4.27-9.21] | **1.59** [1.10-2.48] | **76.73** [47.07-97.48] | **20.84** [11.40-29.88] | **1.35** [0.89-1.92] | **0.36** [0.23-0.49] |
| LUMPY | **209.05** [130.14-318.90] | **54.58** [75.32-37.29] | **13.33** [6.33-22.36] | **4.04** [2.87-5.79] | **13.34** [6.33-22.36] | **4.04** [2.89-5.79] | **5.92** [5.13-6.84] | **1.66** [0.80-2.61] |
| PINDEL | **209.05** [130.14-318.90] | **54.58** [75.32-37.29] | **18.13** [13.63- 27.53] | **5.21** [3.41-7.46] | **20.59** [10.54-35.74] | **5.36** [3.98-6.93] | **9.39** [5.87-12.41] | **2.55** [1.45-3.24] |
| SWAN | **209.05** [130.14-318.90] | **54.58** [75.32-37.29] | **205.15** [155.39- 262.87] | **57.10** [32.63-107.04] | **68.88** [47.14-77.27] | **19.00** [9.86-33.61] | **70.71** [32.14-108.91] | **18.41** [12.74-28.02 |

**Overview of computational performance for seven SV/indel callers**. The mean (bold), minimum, and maximum values are provided for each caller. Ten subjects were randomly selected from the discovery (Disc) and discovery-extension (Disc+ext) phases. The second and third columns provide the file sizes in gigabytes. The fourth and fifth columns provide the average runtime in hours. The sixth and seventh columns provide the peak CPU usage. Columns eight and nine provide the peak memory in gigabytes.
