# Supplementary material for: A comparative study of structural variant calling in WGS from Alzheimer’s disease families
Source: Life Sci Alliance. 2024 Feb 28;7(5):e202302181. doi: 10.26508/lsa.202302181 (PMC10902710; doi:10.26508/lsa.202302181)
Supplement: Supplementary file 2 [file LSA-2023-02181_TableS2.docx]

**Supplementary Tables**

| Supplementary Table 2. Candidate AD Genes |
| --- |
| *ABCA7* |
| *ABCG1* |
| *ABI3* |
| *ACE* |
| *ADAM10* |
| *ADAMTS1* |
| *AKAP9* |
| *APOE* |
| *APP* |
| *BIN1* |
| *BZRAP1* |
| *CASP7* |
| *CASS4* |
| *CD2AP* |
| *CD33* |
| *CELF1* |
| *CLU* |
| *COBL* |
| *CR1* |
| *CTDP1* |
| *ECHDC3* |
| *EPHA1* |
| *FBXL7* |
| *FERMT2* |
| *FRMD4A* |
| *GALNT7* |
| *GAS2L2* |
| *GCH1* |
| *GLIS1* |
| *GLIS3* |
| *GPAA* |
| *HBEGF* |
| *HDAC9* |
| *IGHV1-67* |
| *INPP5D* |
| *IQCK* |
| *KANSL1* |
| *KCNJ15* |
| *KCNMB2* |
| *MAPT* |
| *MEF2C* |
| *MS4A4A* |
| *MS4A6A* |
| *NCR2* |
| *NME8* |
| *NOTCH3* |
| *OPRL1* |
| *OSBPL6* |
| *OSTN* |
| *PCDH8* |
| *PDCL3* |
| *PICALM* |
| *PILRA* |
| *PLCG2* |
| *PLD3* |
| *PLXNA4* |
| *PSEN1* |
| *PSEN2* |
| *PTK2B* |
| *PTPRG* |
| *SERPINB1* |
| *SHARPIN* |
| *SLC10A2* |
| *SLC24A4* |
| *SORL1* |
| *TM2D3* |
| *TP53INP1* |
| *TPBG* |
| *TREM2* |
| *TRIP4* |
| *TTC3* |
| *UNC5C* |
| *ZCWPW1* |
| *ZNF655* |
