# Supplementary material for: A comparative study of structural variant calling in WGS from Alzheimer’s disease families
Source: Life Sci Alliance. 2024 Feb 28;7(5):e202302181. doi: 10.26508/lsa.202302181 (PMC10902710; doi:10.26508/lsa.202302181)
Supplement: Supplementary file 6 [file LSA-2023-02181_TableS6.docx]

**Supplementary Tables**

| **Supplementary Table 6. Deletion Size Frequency Distribution** | | | | | | |
| --- | --- | --- | --- | --- | --- | --- |
|  |  |  |  |  | |  |
| Bin Size | Scalpel | | Parliament |  | Comments | |
| 20-99 | 82,180 | 88.69% | 2,253 | 10.13% | |  |
| 100-199 | 3,984 | 4.30% | 6,601 | 29.67% | |  |
| 200-299 | 1,502 | 1.62% | 2,455 | 11.04% | |  |
| 300-399 | 2,745 | 2.96% | 3,195 | 14.36% | | ALU Peak |
| 400-499 | 662 | 0.71% | 982 | 4.41% | |  |
| 500-599 | 518 | 0.56% | 730 | 3.28% | |  |
| 600-699 | 429 | 0.46% | 529 | 2.38% | |  |
| 700-799 | 376 | 0.41% | 359 | 1.61% | |  |
| 800-899 | 242 | 0.26% | 257 | 1.16% | |  |
| 900-999 | 21 | 0.02% | 221 | 0.99% | |  |
| 1,000-1,999 | NA |  | 1,321 | 5.94% | | SVA Peak |
| 2,000-2,999 | NA |  | 855 | 3.84% | |  |
| 3,000-3,999 | NA |  | 572 | 2.57% | |  |
| 4,000-4,999 | NA |  | 403 | 1.81% | |  |
| 5,000-9,999 | NA |  | 983 | 4.42% | | L1 Peak |
| 10,000-99,999 | NA |  | 530 | 2.38% | |  |
| Total* | 92,659 | 100% | 22,246 | 100% | |  |
|  |  |  |  |  | |  |
| * Singletons excluded | |  |  |  | |  |
